# Epidemiology and primary location of melanoma in Asian patients: A surveillance, epidemiology, and end result–based study

**DOI:** 10.1016/j.jdin.2024.04.006

**Published:** 2024-04-24

**Authors:** Alim Osman, Alexandra R. Nigro, Sarah F. Brauer, Luis J. Borda, Alice A. Roberts

**Affiliations:** a School of Medicine, Eastern Virginia Medical School, Norfolk, Virginia; b Department of Dermatology, Eastern Virginia Medical School, Norfolk, Virginia

**Keywords:** Asian, incidence, malignant melanoma, melanoma, prevalence, SEER

*To the Editor:* The incidence of melanoma is increasing among all racial and ethnic groups, especially in Asians, with an estimated incidence of 1.3 per 100,000.^1^ In contrast to their Caucasian counterparts, Asian patients with melanoma have a 27% higher mortality rate.^2^ In light of the rapidly changing demographic dynamics occurring within the US population, there is a need to describe the epidemiologic nuances of melanoma specifically within the Asian demographic and juxtapose these findings with those observed in other racial groups. Therefore, the purpose of our study is to further investigate melanoma epidemiology disparities among the Asian population.

The Surveillance, Epidemiology, and End Result (SEER) database was analyzed for all melanoma cases from 1975 to 2020. The SEER-22 registry was used, representing 47.8% of the US population. The following racial groups were analyzed: Asian/Pacific Islanders (API), White, Black, and Native Americans (NA). The variables collected include age of diagnosis, sex, and primary site. In this study, sex was defined as the biological difference between males and females. Categorical variables were reported as counts and proportions. A 2-sample *Z* test was used to assess the association between categorical variables. Statistical analysis was conducted using Microsoft Excel. *P* values of <.05 were considered statistically significant.

A total of 196,778 patients with melanoma were identified, of which 1569 (0.8%) were API. API patients were older than NA patients (age ≥ 70 years, 32.2% vs 22%, respectively; *P* < .001; Table I). No sex predilection was found in API patients (50.1% male) whereas White patients were predominantly male (56.3%, *P* < .001). The most common location of melanoma within the API group was the lower limb/hip (*n* = 575, 36.6%), followed by the trunk (*n* = 364, 23.1%) and the upper limb/shoulder (*n* = 303, 19.3%). Furthermore, melanoma of the lower extremity was more commonly found in Asian patients than White and NA patients (36.6% vs 18.2% vs 20.8%, respectively; *P* < .001), but less frequently than Black patients (36.6% vs 48.38%; *P* < .001).

**Table I:** Demographics and primary site of patients with melanoma

Characteristic	Patients, no. (%)				*P* value
	White (*n* = 189,870)	Black (*n* = 710)	NA (*n* = 518)	API (*n* = 1569)	
Age range (y)					<.001 (API vs NA)
≥70	57,882 (30.5)	227 (32)	114 (22)	505 (32.2)	
<70	131,988 (69.5)	483 (68)	404 (88)	1,064 (67.8)	
Sex					<.001 (White and API)
Female	82,890 (43.7)	359 (50.6)	257 (49.6)	783 (49.9)	
Male	106,980 (56.3)	351 (49.4)	261 (50.4)	786 (50.1)	
Melanoma primary site					
Lower limb/hip	34,645 (18.2)	344 (48.5)	108 (20.8)	575 (36.6)	<.001 (API)
Upper limb/shoulder	46,790 (24.6)	102 (14.4)	104 (20.1)	303 (19.3)	
Trunk	61,655 (32.5)	109 (15.4)	160 (30.1)	364 (23.2)	
Scalp/neck	14,672 (7.7)	28 (3.9)	42 (8.1)	70 (4.5)	
Other∗	32,108 (16.9)	127 (17.9)	104 (20.1)	257 (16.4)	

*API*, Asian/Pacific Islander; *NA*, Native American.

∗ Skin of lip, eyelid, external ear, skin other/unspecified, overlapping region, not otherwise specified.

Compared with NA patients, API patients presented with melanoma at an older age and more commonly on the lower extremities compared with NA and White patients. Asian patients have an increased likelihood of behavioral adaptations to sun exposure such as seeking shade and wearing protective clothing, although Asian patients are less likely to receive total body skin examinations and wear sunscreen when compared with White individuals, given the misconception that they are immune to skin cancer which could contribute to melanomas being discovered at older ages in this racial group.^3^^,^^4^

Moreover, the most common reported site of melanoma in Asian patients is the palms, soles, and nails,^5^ however our findings indicate that the lower limb/hip was the most common site. In many Asian cultures, traditional clothing often covers most of the body, leaving the legs exposed, leading to the observed increased rates of leg melanoma in our cohort.

Strengths of this study includes its large populations size in the SEER-22 registry compared with previous studies. Limitations include the retrospective design of SEER, not being able to cover the entire US population, different melanoma subtypes/rates between racial groups, and the potential for underreporting.

## Conflicts of interest

None disclosed.
